# High-throughput phenotyping for climate-resilient forests: integrating multi-sensor fusion and root-shoot dynamics

**DOI:** 10.3389/fpls.2026.1842337

**Published:** 2026-05-20

**Authors:** Jun Lou, Yeqing Peng, Nanxi Lyu, Xijian Fan, Hao Liang, Yanjie Li

**Affiliations:** 1 Forestry Station, Agricultural Technology Promotion Center of Fuyang District, Hangzhou, Zhejiang, China; 2 Matou State-Owned Forest Farm, Jing County, Xuancheng, Anhui, China; 3 Centre for Smart Forestry Innovation and Research, Research Institute of Subtropical Forestry, Chinese Academy of Forestry, Hangzhou, Zhejiang, China; 4 College of Information Science and Technology, Nanjing Forestry University, Nanjing, China; 5 State Key Laboratory of Subtropical Silviculture, Zhejiang Agriculture and Forestry University, Hangzhou, China

**Keywords:** artificial intelligence (AI), climate-smart forestry, digital twin, drought resilience, high-throughput phenotyping

## Abstract

Climate change is increasing the frequency of compound drought and heat events, threatening forest stability worldwide. While genomics has helped identify resilient genotypes, our ability to characterize adaptive traits — phenotyping — has not kept pace. This creates a bottleneck: we can sequence trees faster than we can understand how they physically respond to stress. Moving away from single-sensor monitoring, the field is now embracing multi-sensor data fusion, in which thermal imaging, Solar-Induced Fluorescence (SIF), hyperspectral remote sensing, and LiDAR are combined on platforms ranging from Unmanned Aerial Vehicles (UAVs) to ground-based robotic systems. These integrated approaches are proving effective for detecting physiological stress — such as changes in stomatal conductance — before visible damage appears. Deep learning models, meanwhile, are beginning to outperform traditional vegetation indices for specific tasks such as tree-crown segmentation and stress classification, although their performance remains constrained by overfitting, limited transferability, and domain shift across forest types in analyzing complex forest canopies. A major limitation remains, however: most high-throughput phenotyping (HTP) focuses on the canopy, largely ignoring the root system and the soil–plant–atmosphere continuum (SPAC), which are critical for drought resilience. In this review, we argue that developing climate-resilient forests requires looking below the canopy. We propose a constraint-based framework that couples aerial sensor data with eco-hydrological approaches and process-based modeling to narrow the range of plausible root functional strategies—rather than to directly identify root phenotypes, while critically evaluating the assumptions and validation challenges inherent in this approach. Future research should focus on standardized protocols, open benchmark datasets, and Explainable AI (XAI) to strengthen the link between above-ground signals and below-ground traits.

## 1 Introduction

Forests serve as essential global carbon sinks, playing a central role in slowing anthropogenic climate change (Pan et al., 2011; Werner et al., 2025). Their stability, however, is under increasing threat. Compound drought and heat events — where prolonged rain deficits coincide with severe heatwaves — are growing in frequency and intensity (Zscheischler et al., 2018, Seneviratne, Zhang et al., 2016; Romanou et al., 2025). This combination creates acute physiological stress: atmospheric water demand rises sharply while soil moisture dwindles, pushing trees towards their hydraulic tipping points (Groover et al., 2025). Tree mortality under these conditions is widely hypothesized to involve interacting mechanisms, most commonly hydraulic failure, where gas embolisms block water transport through xylem tissues, and carbon starvation triggered by prolonged stomatal closure, although the relative contribution of each process remains debated (McDowell et al., 2008; Phillips et al., 2016; Groover et al., 2025).

This escalating threat demands a fundamental shift in forestry practices. Traditional silviculture, focused primarily on timber volume, must evolve towards ‘climate-smart forestry,’ which prioritises breeding and deploying genotypes with superior resilience and resource efficiency (Cooper and MacFarlane, 2023). Yet our capacity to identify how different species and genotypes adapt to rapidly changing conditions remains limited by several methodological barriers: the sheer physical size of trees makes whole-organism measurement difficult; physiological responses are transient and spatially heterogeneous across canopies; and long generation times slow breeding cycles (Kremer et al., 2012; Alberto et al., 2013; Feng et al., 2024).

The past decade has produced remarkable advances in forest genomics, enabling researchers to rapidly identify candidate genes linked to stress tolerance (Strauss and Brunner, 2010; Plomion et al., 2016; Li et al., 2024). This genomic progress has, paradoxically, exposed a major operational gap: while genetic markers can be screened across thousands of trees in weeks, characterising their physical and physiological traits — the process known as phenotyping — remains slow, often destructive, and labor-intensive (Bian et al., 2022; Visakh et al., 2024). This disparity, widely termed the “phenotyping bottleneck,” prevents us from translating genomic data into real-world climate resilience (Furbank and Tester, 2011; Hein et al., 2021). Traditional physiological measurements such as leaf water potential or gas exchange provide deep mechanistic insights, but gas exchange measurements are either ecosystem-level ‘black boxes’ (e.g., eddy covariance) or capture only a small portion of a single tree at one point in time (Mansoor and Chung, 2024). These approaches cannot track the dynamic, daily responses of whole stands to fluctuating stress. Without scalable phenotyping, the potential of genomics for climate adaptation will remain largely theoretical (Großkinsky et al., 2015; Sharma et al., 2025).

High-Throughput Phenotyping (HTP) offers a route past this bottleneck. The term encompasses a broad suite of technologies — not limited to drone-based sensors, but extending to ground-based robotic platforms, automated sensor arrays, and high-throughput greenhouse systems — that enable rapid, non-invasive measurement of plant traits across large populations (Araus and Cairns, 2014; Yang et al., 2020; Kim et al., 2021). Originally developed for the relatively uniform canopies of crop science (Liang et al., 2025), HTP is increasingly being adapted for forestry. This transition is far from simple, however. Forests present challenges that crop fields do not: intricate three-dimensional canopy structures, substantial variation in tree height and crown architecture, long life cycles, mixed species composition, and — perhaps most fundamentally — the large physical size and structural complexity of trees, making whole-organism assessment at throughputs comparable to crop phenotyping extremely difficult (da Silva et al., 2021; Ardohain, 2025).

Recent sensor advances are beginning to address some of these complexities. Thermal infrared imaging can map canopy temperature variability non-invasively, serving as a scalable proxy for stomatal conductance and transpiration rates (Torresan et al., 2021; Smigaj et al., 2024). Hyperspectral sensors detect subtle biochemical changes — pigment degradation, shifts in water content — often before visible decline appears (Mahlein et al., 2018; Groover et al., 2025). Solar-Induced chlorophyll Fluorescence (SIF), retrieved from narrow spectral windows, provides a direct window into photosynthetic electron transport efficiency and has proven sensitive to early drought stress in canopies (Mohammed et al., 2019; Porcar-Castell et al., 2021). Emerging microwave remote sensing approaches, meanwhile, offer the ability to penetrate canopy layers and assess vegetation water content and soil moisture simultaneously, though their spatial resolution on airborne platforms remains a challenge (Konings et al., 2019; Moesinger et al., 2022). When these data streams are combined with Light Detection and Ranging (LiDAR) for precise structural metrics, researchers gain an unprecedented ability to monitor forests from individual trees to entire stands (White et al., 2016; Sankey et al., 2017; Torresan et al., 2021). A ‘digital twin’ — that is, a dynamic, predictive virtual replica of a forest stand that continuously integrates real-time sensor data with process-based physiological models — represents the logical culmination of this multi-sensor approach (Das, 2025).

Despite this progress, two critical gaps remain. The first is an integration gap: single-sensor approaches rarely provide a complete picture of drought stress, and the field urgently needs robust multi-sensor data fusion frameworks that go beyond merely overlaying spatial data layers (Ghamisi et al., 2019; Gwal et al., 2026). The second, and arguably more fundamental, is a “canopy bias” in current HTP strategies. Nearly all existing work focuses on above-ground traits while neglecting the root system and its dynamic interactions with soil (Norby and Jackson, 2000; Freschet et al., 2021a). Drought resilience is, at its core, a whole-plant attribute, governed by the coordination between above-ground water regulation (e.g., stomatal control) and below-ground water acquisition (e.g., root architecture, mycorrhizal associations, hydraulic redistribution) (Fan et al., 2017; Brodribb et al., 2020). A tree may appear ‘resilient’ in aerial imagery not because of superior physiological efficiency per se, but because it possesses a deeper root system accessing stable groundwater — an adaptive trait invisible to canopy-level sensors (Fan et al., 2017).

To address these gaps, this review synthesises state-of-the-art HTP technologies specifically for forest climate resilience. Unlike previous reviews that have comprehensively covered remote sensing advances for vegetation monitoring (Kattenborn et al., 2021; Bian et al., 2022; Smigaj et al., 2024), our focus is on the relatively under-explored intersection of multi-sensor fusion with whole-plant physiology, particularly the integration of above- and below-ground traits. We first briefly review sensor technologies and AI approaches (Section 3), then devote extended treatment to the challenge and potential of bridging aerial signals and subterranean root function (Section 4), including a critical evaluation of the assumptions, limitations, and validation pathways involved. This paper is a semi-systematic review combining structured database searching with narrative synthesis. The literature reviewed here was identified through systematic searches of Web of Science and Scopus (January 2015–December 2025), using Boolean combinations of keywords in title/abstract/keyword fields including “high-throughput phenotyping,” “forest resilience,” “UAV thermal,” “hyperspectral forestry,” “solar-induced fluorescence,” “multi-sensor fusion,” “deep learning forestry,” and “root phenotyping.” An example full search string was: (“high-throughput phenotyping” OR “HTP”) AND (forest* OR tree* OR woody) AND (drought OR heat OR “climate resilience”). Inclusion criteria were: (i) peer-reviewed original research articles; (ii) application to woody vegetation or forest stands; (iii) use of remote sensing or HTP methods; and (iv) assessment of physiological, structural, or functional traits relevant to abiotic stress. Exclusion criteria were: non-English records, conference abstracts without full text, purely methodological papers lacking ecological or physiological application, and studies restricted to herbaceous crops. An initial pool of 417 records was screened at title/abstract level (213 removed), leaving 204 for full-text assessment; a further 114 were excluded for failing to meet the inclusion criteria. A total of 90 original research articles were retained and form the core empirical basis for our synthesis.

## 2 Trends in high-throughput forest phenotyping research

**Figure 1:**
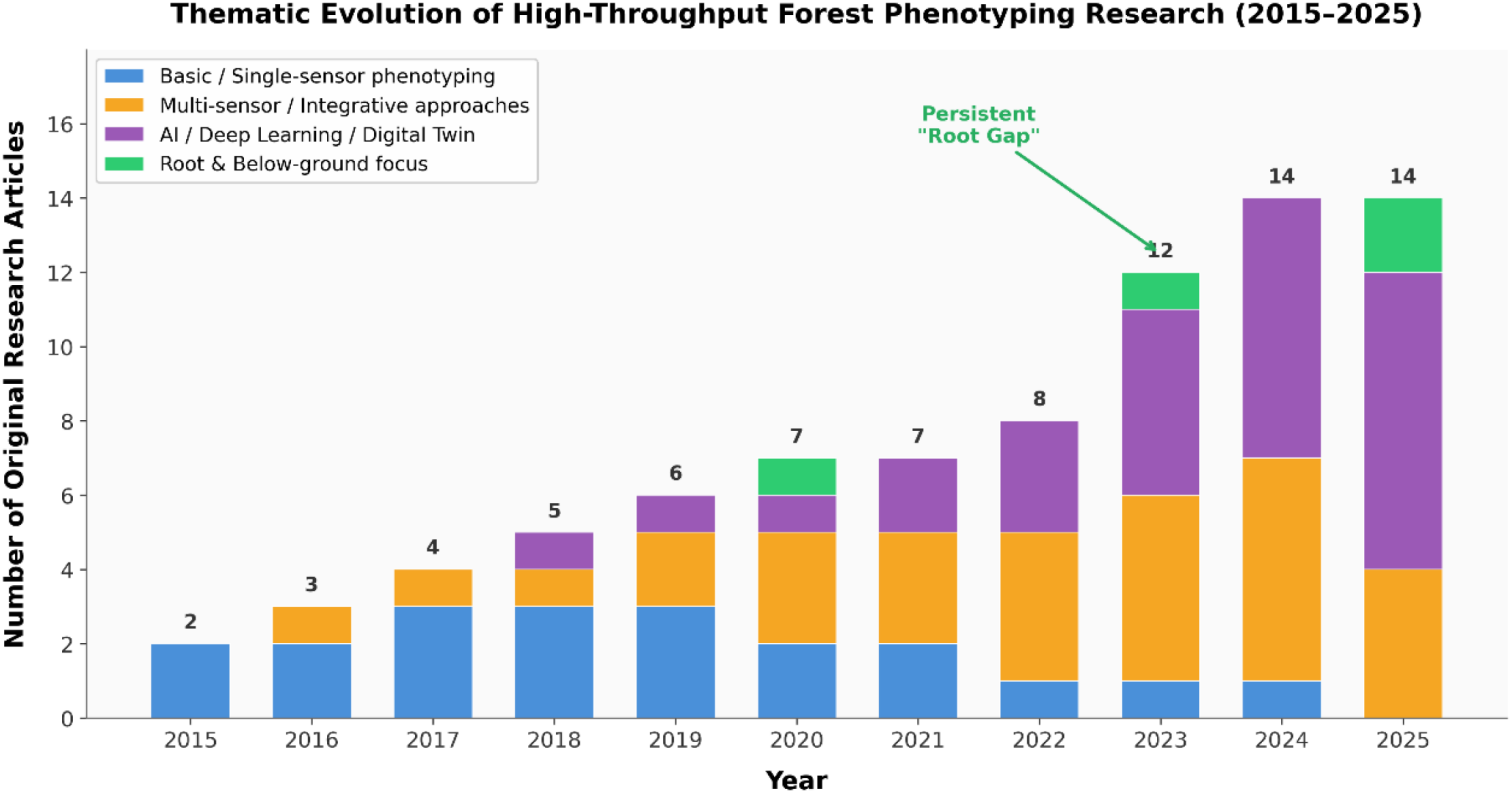
Thematic evolution of original empirical research in high-throughput forest phenotyping, 2015–2025 (review articles and perspectives excluded). Studies are classified along two dimensions: (i) methodological complexity — basic/single-sensor (blue), multi-sensor/integrative (orange), and AI/deep learning/digital twin (purple); and (ii) plant component focus, with studies explicitly addressing root and below-ground traits indicated in green. Categories are defined in the legend: blue = single-sensor phenotyping (e.g., individual thermal or spectral index validation); orange = multi-sensor or integrative approaches combining two or more data streams; purple = studies employing AI, deep learning, or digital-twin frameworks; green = studies with explicit root or below-ground focus (any methodological category).

A statistical overview of the selected empirical research reveals clear patterns over the past decade (2015–2025; **Figure 1**). The volume of original research has increased steadily, with a marked acceleration from 2023 to 2025, coinciding with the maturation of UAV platforms and the operational deployment of advanced sensors in field settings. The thematic composition has also shifted substantially. Early experimental work (2015–2018) centred largely on basic single-sensor phenotyping — primarily validating individual thermal or spectral indices. From 2021 onwards, and peaking in 2024–2025, studies using multi-sensor fusion and AI/deep learning approaches have come to dominate the empirical literature, reflecting a transition from data collection to complex, high-dimensional data analysis.

Alongside this methodological evolution, **Figure 1** reveals a persistent and critical research gap. To make this gap visible, we categorised studies along two independent dimensions: methodological complexity (single-sensor, multi-sensor/integrative, or AI/deep learning/digital twin) and plant component focus (above-ground only, below-ground only, or integrated above- and below-ground). Studies explicitly addressing root and below-ground processes remain a strikingly small proportion of the literature across all years. Even within the recent surge in advanced computational approaches, root-focused investigations appear only sporadically (minor presence in 2020, 2023, and 2025) and are entirely absent from several years’ records (2018, 2019, 2021, 2022, and 2024). This disparity — above-ground AI applications flourishing while below-ground traits remain ‘hidden’ — underscores the necessity of the holistic phenotyping framework developed in Section 4 of this review.

It is worth noting, however, that the small absolute number of publications addressing below-ground traits (reflecting the overall small corpus of HTP forestry studies) means that the ‘sporadic’ appearance of root-focused work may partly reflect the general immaturity of the field rather than systematic neglect. Above-ground remote sensing is more technically advanced than below-ground sensing; data fusion approaches for the subsurface are therefore naturally lagging. Nevertheless, the near-complete absence of root-focused studies in most years suggests that a genuine gap exists beyond what sample-size effects alone can explain.

## 3 Sensor technologies, data fusion, and AI for forest phenotyping

The transition from conventional forestry surveys to high-throughput physiological phenotyping depends on sensors capable of detecting stress signals outside the visible spectrum. This section provides a condensed overview of the principal sensing modalities and analytical approaches; readers seeking comprehensive technology reviews are directed to recent syntheses (Lausch et al., 2016; Kattenborn et al., 2021; Sun et al., 2021; Bian et al., 2022; Smigaj et al., 2024). Our aim here is to establish the technical foundation necessary for the integrative root-shoot framework developed in Section 4.

### 3.1 Thermal, spectral, and fluorescence sensing

Thermal infrared (TIR) imaging exploits the leaf energy balance: under adequate water supply, transpirational cooling keeps leaf temperatures below ambient air temperature, whereas stomatal closure under water deficit reduces latent heat loss and elevates canopy temperature (Tc) (Jones et al., 2002; Smigaj et al., 2024). UAV-based thermography can map these temperature variations at individual-tree or stand scale, offering a non-invasive alternative to manual porometry (Ludovisi et al., 2017; Torresan et al., 2021). Metrics such as Canopy Temperature Depression (CTD) and the Crop Water Stress Index (CWSI) have been successfully adapted from agriculture to forestry for ranking genotypes by water-use efficiency and stomatal regulation strategy (Araus et al., 2018; Italiano et al., 2023). Detecting isohydric behaviour — where trees rapidly close stomata to maintain constant water potential — is particularly valuable, as this trait is imperceptible visually but clearly expressed in the thermal spectrum (Rowland et al., 2023). The chief complication in forests, compared with uniform crop canopies, is surface roughness: complex aerodynamics, varied sun-canopy-sensor geometries, and heterogeneous background temperatures (bare soil, understory) all introduce noise into thermal data (Aubrecht et al., 2016; Stickley and Fraterrigo, 2021). Current mitigation strategies include normalisation against empirical ‘wet’ and ‘dry’ reference surfaces and integration of thermal data with LiDAR-derived 3D structural models to correct for shading and illumination effects (Hernández-Clemente et al., 2019).

Spectral sensors (multispectral and hyperspectral) capture biochemical and metabolic signatures reflecting longer-term physiological adjustments to stress (Lausch et al., 2016; Ustin and Jacquemoud, 2020). Drought and heat stress alter leaf pigment concentrations, cell structure, and water content, producing characteristic changes in canopy reflectance across distinct wavebands (Ollinger, 2011). While broadband indices such as NDVI remain widely used for general health monitoring, they saturate in dense canopies and typically lag behind physiological stress onset (Jangra et al., 2021). Narrow-band hyperspectral indices offer greater precision: the Photochemical Reflectance Index (PRI) tracks xanthophyll-cycle dynamics as an early indicator of photosynthetic downregulation (Peguero-Pina et al., 2008; Zhang et al., 2016), while water-sensitive SWIR indices correlate with leaf and canopy water potential (Colombo et al., 2008; Cotrozzi et al., 2017). A persistent challenge is identifying robust spectral signatures that generalise across species, developmental stages, and environmental conditions — a problem exacerbated by the high dimensionality and collinearity inherent in hyperspectral data (Hennessy et al., 2020; Schratz et al., 2021).

Solar-Induced chlorophyll Fluorescence (SIF) represents a particularly promising, yet still emerging, data stream for forest HTP. SIF is emitted by chlorophyll during photosynthesis and can be retrieved from narrow spectral windows in the oxygen absorption bands. Unlike reflectance-based indices that respond to changes in pigment concentration or canopy structure, SIF provides a more direct measure of actual photosynthetic electron transport activity (Mohammed et al., 2019; Porcar-Castell et al., 2021). This makes SIF potentially more sensitive to the earliest stages of drought stress, when photosynthetic machinery is downregulated but leaf biochemistry has not yet visibly changed. The deployment of SIF retrieval on UAV and airborne platforms for forest applications is still in its early stages, and significant challenges remain in atmospheric correction, signal-to-noise ratios over heterogeneous canopies, and disentangling SIF from reflected radiance (Köhler et al., 2018). Nonetheless, SIF adds a fundamentally different dimension of physiological information that complements thermal and reflectance data.

It is important to note that the sensors described above do not exhaust the HTP toolkit. Microwave remote sensing, for instance, can penetrate canopy layers to assess vegetation water content and soil moisture (Gevaert et al., 2016; Moesinger et al., 2022), and emerging radar approaches on airborne platforms offer potential for monitoring forest water dynamics at scales intermediate between ground measurements and satellite observations (Konings et al., 2019). A summary of the principal sensing modalities, the physiological information they capture, and their key limitations is presented in **Table 1**.

**Table 1:** Summary of principal sensing modalities for forest high-throughput phenotyping, the physiological traits they capture, and key limitations.

Sensing modality	Platform (s)	Primary physiological information	Key limitations
Thermal infrared (TIR)	UAV, ground-based	Stomatal conductance (via Tc), transpiration, water stress	Sensitive to wind, solar radiation, canopy geometry; requires normalisation
Multispectral	UAV, satellite	General canopy health (NDVI, etc.), broad pigment changes	Saturates in dense canopies; limited biochemical specificity
Hyperspectral	UAV, airborne, ground	Pigment composition, water content, photosynthetic efficiency (PRI)	High dimensionality; collinearity; species-specific calibration needed
Solar-Induced Fluorescence (SIF)	Airborne, tower, emerging UAV	Photosynthetic electron transport activity	Atmospheric correction challenges; low signal-to-noise; early-stage deployment
LiDAR	UAV, airborne	3D canopy structure (height, crown volume, LAD)	No direct physiological information; point density dependent
Microwave/Radar	Satellite, airborne	Vegetation water content, soil moisture	Coarse spatial resolution on most platforms; complex signal interpretation
Ground Penetrating Radar (GPR)	Ground-based	Coarse root architecture, soil structure	Small footprint; soil-type dependent; not high-throughput
Electrical Resistivity Tomography (ERT)	Ground-based	Soil moisture dynamics around root zones	Labour-intensive installation; limited spatial coverage; not measuring roots directly

### 3.2 Multi-sensor data fusion

No single sensor captures the full complexity of drought stress in forests. Multi-sensor data fusion aims to combine information from distinct sensing modalities synergistically — not merely overlaying spatial data layers, but integrating complementary information to reduce uncertainty and enhance inference accuracy regarding physiological and ecological processes (Schmitt and Zhu, 2016; Ghamisi et al., 2019). The literature distinguishes three levels of fusion: raw data level (combining sensor outputs before feature extraction), feature level (fusing extracted features from each source), and decision level (integrating independent model outputs) (Pohl and Van Genderen, 1998; Seneviratne et al., 2021).

**Figure 2:**
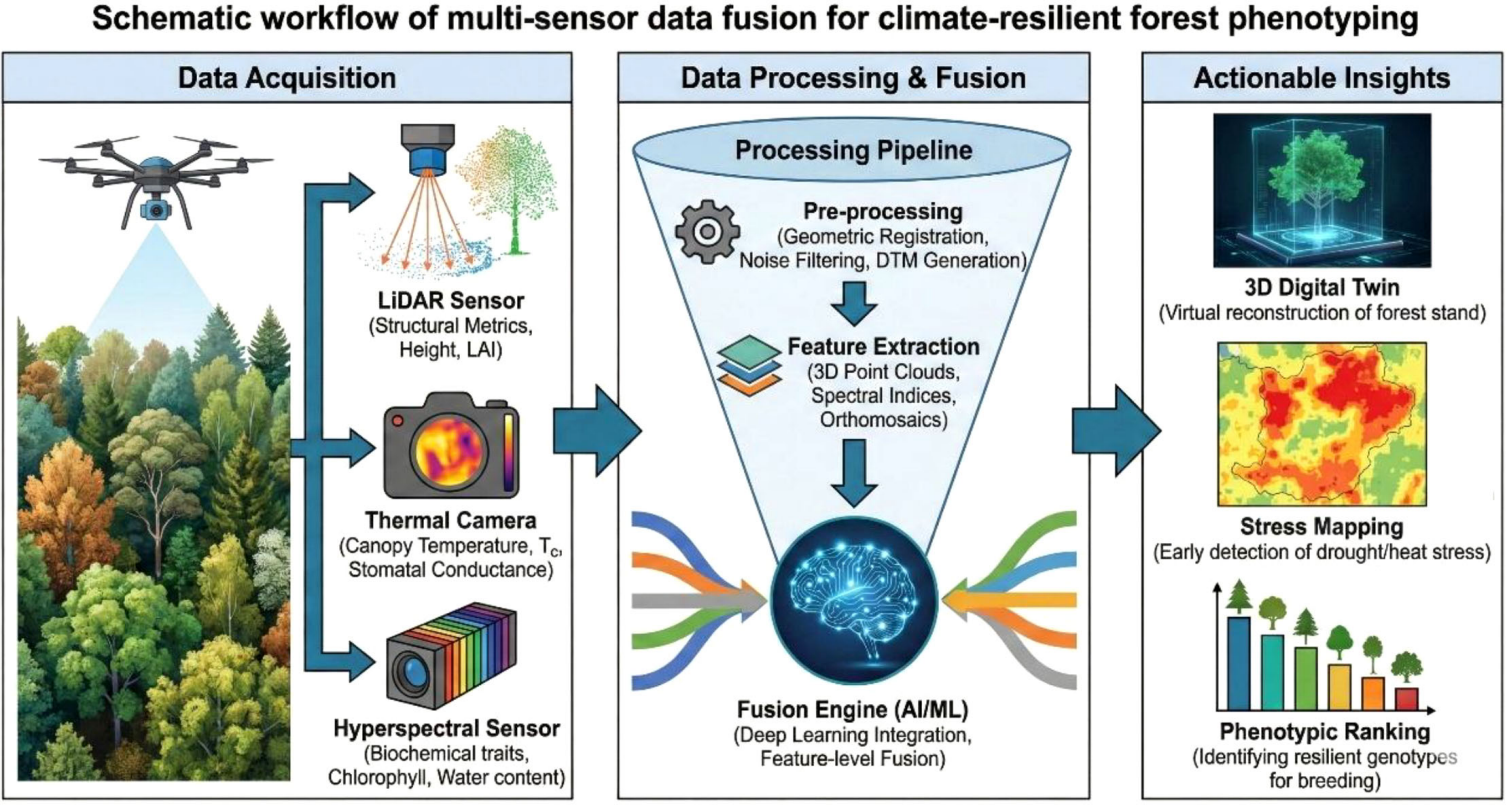
Schematic workflow of integrated multi-sensor data fusion for climate-resilient forest phenotyping. Structural (LiDAR), physiological (Thermal), biochemical (Hyperspectral), and photosynthetic (SIF) data are acquired via UAV and/or ground-based platforms, pre-processed and features extracted, then integrated through an AI-driven fusion engine to generate actionable outputs including 3D digital twins, stress maps, and phenotypic rankings for breeding selection.

For forest phenotyping, the fusion of LiDAR with thermal and/or hyperspectral data has proven particularly powerful. LiDAR provides 3D canopy architecture — tree height, crown volume, Leaf Area Density (LAD) — which is essential for correcting passive optical data for shading, illumination geometry, and canopy structural confounds (Koetz et al., 2007; Tusa et al., 2019). Accurately partitioning ‘sunlit’ and ‘shaded’ leaf area using LiDAR point clouds, for example, substantially improves the robustness of thermal-based stomatal conductance estimates (Jones et al., 2002; Kullberg et al., 2017). When detailed hyperspectral indices (biochemical status) are fused with precise structural traits (from LiDAR), the resulting dataset can feed into dynamic digital twin frameworks that simulate stress responses under varying climate scenarios (Asner et al., 2017; Das, 2025). The conceptual workflow for multi-sensor fusion is illustrated in **Figure 2** (conceptual schematic). A practical complication, often underappreciated, is that the sensors being fused do not operate at matched spatial or temporal scales. Thermal imaging typically captures whole-canopy temperatures at sub-hourly frequencies, hyperspectral instruments resolve leaf- to crown-level biochemistry but on slower revisit cycles, SIF is retrieved at footprint scales of metres to tens of metres, and LiDAR point clouds are usually acquired only once or a few times per growing season. These scale mismatches complicate direct pixel-wise fusion: features that are meaningful at leaf scale may be diluted at crown scale, while signals that are ecologically interpretable at stand scale may mask within-tree heterogeneity. Robust fusion therefore requires either explicit upscaling and downscaling steps, or model architectures (e.g., attention-based networks) capable of reconciling data streams acquired at different resolutions.

### 3.3 AI and deep learning

**Figure 3:**
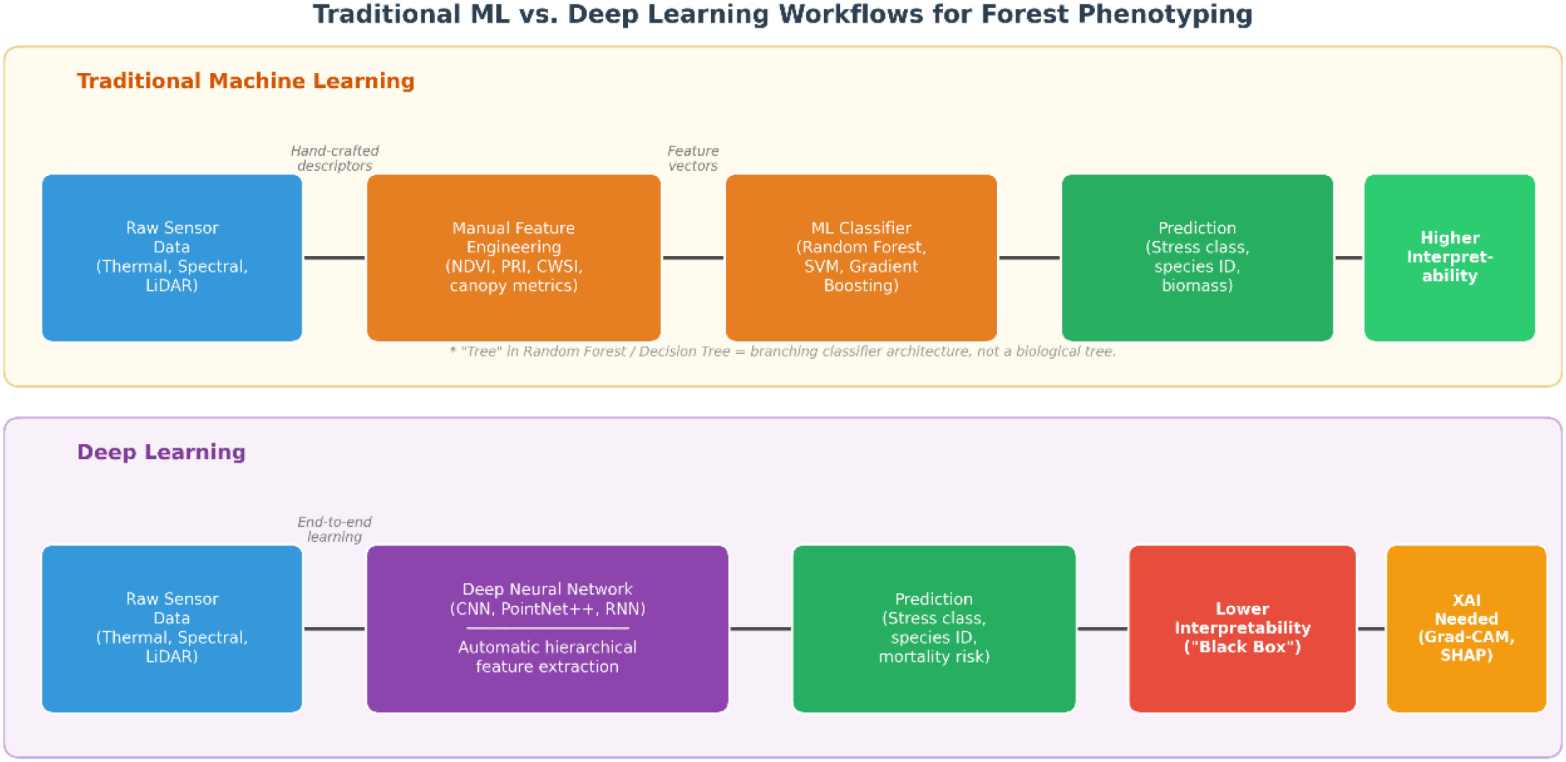
Conceptual comparison of workflows between traditional machine learning (ML) and deep learning (DL) for forest phenotyping tasks. Traditional ML relies on manual feature engineering, whereby researchers design hand-crafted descriptors from sensor data, feed them into classifiers (e.g., Random Forest, SVM), and obtain predictions. DL utilises end-to-end learning to automatically extract hierarchical features from raw data through successive network layers. The “tree” structure in the ML pathway refers to the branching architecture of a decision tree classifier, not to a biological tree.

The integration of multi-modal sensors generates massive, heterogeneous datasets that exceed the capacity of traditional statistical analyses (Chi et al., 2016). Machine Learning (ML) and Deep Learning (DL) algorithms have become indispensable for extracting meaningful patterns from these data (Zhu et al., 2017, Kamilaris and Prenafeta-Boldú, 2018). Traditional ML approaches — Random Forest (RF), Support Vector Machines (SVM), gradient boosting — remain valuable for tasks where training data are limited and interpretability is important, such as distinguishing stressed from healthy trees based on spectral or structural metrics (Belgiu and Drăguţ, 2016, Maxwell et al., 2018). DL approaches, particularly Convolutional Neural Networks (CNNs) and point-based models such as PointNet++, have demonstrated improved accuracy over traditional ML baselines (typically reported as higher F1, IoU, or overall accuracy on benchmark datasets) in tasks such as semantic segmentation of individual tree crowns, high-resolution disease detection, and prediction of drought-induced mortality from temporal sequences of HTP data (Qi et al., 2017; Feng et al., 2021; Kattenborn et al., 2021). The key advantage of DL is automatic extraction of hierarchical features from raw image and point-cloud data, reducing the need for manual feature engineering — that is, the labour-intensive process of designing hand-crafted descriptors that traditional ML requires (Zhu et al., 2017). A comparative overview of these approaches is presented in **Table 2** and **Figure 3** (both conceptual schematics). It is important to emphasise that reported DL superiority is typically established on curated benchmarks and can degrade sharply in operational settings. Persistent issues include overfitting on small training sets, limited transferability between forest types and sensors, class imbalance in stress detection (healthy pixels vastly outnumber stressed ones), domain shift between training and deployment sites, and a general scarcity of annotated labels for forest HTP compared with agricultural or general-purpose vision benchmarks.

**Table 2:** Comparative analysis of traditional ML and DL algorithms applied in forest phenotyping and stress detection.

Algorithm class	Specific models	Key advantages	Limitations	Typical applications in forestry	Key references
Traditional Machine Learning	Random Forest (RF)	High interpretability; handles noise well; effective on smaller datasets typical of field trials	Requires manual feature engineering; performance plateaus with massive datasets	Semantic segmentation (wood/leaf separation); Biomass estimation	(Belgiu and Drăguţ, 2016, Maxwell et al., 2018; Nogueira et al., 2023; Gou et al., 2024)
	Support Vector Machine (SVM)	Effective in high-dimensional feature spaces; robust against overfitting in small samples	Computationally expensive for large point clouds; slow training speed	Tree species classification; Vertical structure analysis	(Mountrakis et al., 2011; Maraveas, 2024)
Deep Learning	Convolutional Neural Networks (CNN)	Automatic feature learning from raw data; state-of-the-art accuracy in image tasks	Low interpretability (‘black box’); requires large annotated training datasets	Disease/damage detection; Early stress symptom identification from imagery	(Kattenborn et al., 2021; Liu and Wang, 2021; Kumari et al., 2025
	Point-Based Models (e.g., PointNet++)	Processes raw 3D point clouds directly; captures local geometric structures efficiently	Computationally intensive; requires consistent point density	Individual tree segmentation; 3D species classification from LiDAR	(Qi et al., 2017; Briechle et al., 2020; Liu et al., 2022, Liu et al., 2024

### 3.4 Challenges and the imperative for explainable AI

A critical barrier to operational deployment of AI in forestry HTP is the ‘black box’ nature of many DL models. In forestry, where understanding the biological drivers of stress and resilience is paramount — not merely predicting outcomes but informing management and breeding decisions — lack of interpretability can hinder scientific understanding and practitioner acceptance (Maxwell et al., 2018; Reichstein et al., 2019).

Explainable AI (XAI) encompasses a suite of techniques designed to make model predictions interpretable. Gradient-weighted Class Activation Mapping (Grad-CAM), for instance, highlights which spatial regions of an input image most strongly influenced a CNN’s prediction, potentially revealing which canopy features the model associates with drought stress. SHAP (SHapley Additive exPlanations) values quantify the contribution of each input feature to individual predictions, enabling researchers to verify whether a model’s reasoning aligns with known plant physiology. Layer-wise Relevance Propagation (LRP) traces prediction contributions back through network layers. These techniques have seen growing application in ecological remote sensing (Reichstein et al., 2019), though their adoption in forest phenotyping specifically remains limited.

The question of whether XAI can meaningfully bridge the gap between statistical prediction and biological understanding is not yet settled, however. Many XAI methods provide *post-hoc* rationalisation rather than true mechanistic insight; a model may achieve high accuracy through correlations that are biologically spurious or confounded by environmental covariates. It is also worth noting the limits of XAI itself: feature importance does not imply biological causation, Grad-CAM heatmaps and SHAP values can be unstable across runs or seeds, and sensitivity to small input perturbations can make interpretations misleading. For these reasons, XAI should be viewed as a useful diagnostic rather than a substitute for empirical validation—independent below-ground measurements, not interpretability tools, ultimately decide whether a model’s predictions reflect real physiological mechanisms. The development of inherently interpretable architectures — models whose structure embeds domain knowledge (e.g., physics-informed neural networks that encode known SPAC relationships) — may ultimately prove more valuable for forest phenomics than purely *post-hoc* explanations. This remains an active area of research across Earth system science (Reichstein et al., 2019).

A second, compounding challenge is data scarcity. Standardised, open-access benchmark datasets for forest phenomics are largely absent, limiting the generalisability and transferability of AI models across forest types, species, and regions (Araus et al., 2018; Weinstein et al., 2019). The high cost of accurate manual annotation of training data — particularly for tasks like individual tree stress classification, where ground-truth requires laborious field measurements — further constrains model development. Corrective annotation workflows, in which initial model predictions are refined through iterative expert review, offer one promising route to expand training datasets more efficiently (Weinstein et al., 2019). Future progress will depend on both methodological advances in XAI and the establishment of global data-sharing protocols and curated benchmark datasets.

## 4 Bridging the “hidden half”: integrating above- and below-ground phenotypes

Current high-throughput phenotyping strategies in forestry exhibit a significant ‘canopy bias.’ Aerial platforms provide high-resolution views of above-ground morphology and leaf-level physiological responses, but they critically neglect the ‘hidden half’ of the tree: the root system and its interactions with the soil (Eshel and Beeckman, 2013; Freschet et al., 2021b). This is not merely a methodological convenience but a fundamental limitation in understanding drought resilience, which is governed by the dynamic coordination between above-ground water regulation and below-ground water acquisition (Mencuccini, 2003; Brodribb et al., 2020). This section critically evaluates the potential and limitations of bridging this gap. Two clarifications are warranted at the outset. First, we distinguish between structural root traits (e.g., rooting depth, root biomass, branching architecture) and functional root traits (e.g., water uptake dynamics, root hydraulic conductance, water-source partitioning); these are related but not equivalent, and different sensing approaches inform them to different degrees. Throughout this section we aim to specify which class of trait is being inferred. Second, we adopt a constraint-based framing: aerial and eco-hydrological signals are best interpreted as probabilistic indicators that narrow the range of plausible root functional strategies for a given tree or stand, rather than as direct operational measurements of root phenotypes. This framing is central to how we judge the methods discussed below.

### 4.1 The challenge of forest root phenotyping

Characterising root systems in mature forest settings is notoriously difficult. Forest soils are heterogeneous, often rocky, and interlaced with roots from multiple competing species (Bayala and Prieto, 2020). Traditional methods — destructive soil coring and whole-tree excavation — are labour-intensive, destructive, and incapable of capturing how root systems respond dynamically to fluctuating stress over time (Freschet et al., 2021a). Non-destructive proximal technologies such as minirhizotrons and Ground Penetrating Radar (GPR) provide valuable *in-situ* data, but their small measurement footprints make them unsuitable for the stand-scale throughput required in modern breeding programmes (Barton and Montagu, 2004; Zou et al., 2020).

It is important, however, to recognise that the measurement arsenal is broader than these physical methods suggest. A substantial body of eco-hydrological research has developed inferential approaches to root function that complement — and in some cases surpass — direct physical measurement in terms of scalability. Soil moisture dynamics measured at plot and landscape scales can be inverted to estimate effective rooting depth distributions, because patterns of soil water depletion encode information about where roots are actively extracting water (Feldman et al., 2023; Stocker et al., 2023). Stable isotope and tracer techniques (δ^18^O, δ^2^H) can identify the depth and source of water taken up by individual trees, providing species-level information on rooting strategies without excavation (Bachofen et al., 2024). These eco-hydrological approaches yield functional insights into root water uptake patterns at scales relevant to forest management, bridging the gap between point-scale minirhizotron observations and the stand-scale phenotyping ambition of HTP.

Even so, a profound methodological disconnect remains. None of these methods — physical, geophysical, or eco-hydrological — currently operates at the throughput, spatial coverage, and temporal resolution achieved by aerial canopy sensing. This asymmetry motivates the inferential approach discussed next: can above-ground signals, captured at high throughput by UAVs, encode sufficient information about below-ground root function to be operationally useful?

### 4.2 Inferring root function from aerial signals: potential and limitations

The conceptual basis for inferring below-ground traits from canopy observations rests on the integral hydraulic connection of the Soil-Plant-Atmosphere Continuum (SPAC) (Brodribb et al., 2020). Within this continuum, changes in root function translate into altered water transport and, ultimately, observable shifts in canopy physiology. Two inferential pathways have been proposed:

Thermal signatures as proxies for rooting depth and water uptake. Trees with deeper, more extensive, or more hydraulically efficient root systems can access water from deeper soil layers during surface drought, sustaining transpiration longer and maintaining cooler canopy temperatures than their shallow-rooted neighbours (Jones, 2004; Ludovisi et al., 2017). Longitudinal UAV thermal monitoring during natural dry-down events could therefore serve as a high-throughput, indirect identifier of genotypes with superior root water uptake capacity.

Spectral signals and root-shoot allocation trade-offs. Chronic water stress can induce shifts in biomass allocation favouring root growth at the expense of shoot growth. While UAVs cannot directly quantify root biomass, the resulting above-ground trade-offs — reduced Leaf Area Index (detectable by LiDAR) or altered leaf biochemistry (detectable by hyperspectral sensors) — might signal below-ground reallocation of resources (Zarco-Tejada et al., 2012; Homolová et al., 2013).

Although direct empirical attempts to infer root traits from aerial signals in forests remain scarce, a handful of partial demonstrations illustrate what is currently achievable and where the main difficulties lie. In orchard and agroforestry settings, UAV thermal imaging during progressive soil dry-down has been used to rank trees by stomatal sensitivity and indirectly by access to deeper water sources, with rankings broadly consistent with isotope-based water-source measurements at a subset of trees (Gonzalez-Dugo et al., 2013). Airborne hyperspectral and thermal campaigns in Mediterranean and semi-arid woodlands have been combined with modelled root-zone water storage and satellite-derived soil moisture to identify stands whose canopy behaviour is consistent with deeper effective rooting (Meerdink et al., 2025). Joint airborne SIF and thermal measurements have been used to attribute canopy cooling during drought to sustained transpiration rather than hydraulic failure, which in turn implies continued root water supply (Shan et al., 2019). And in a small number of experimental plantations, UAV time series have been cross-referenced with minirhizotron or destructive root-core data at selected trees; these studies typically find statistically meaningful but modest correlations, with substantial unexplained variance attributable to the confounds detailed below. Taken together, these examples support the view that aerial signals can serve as candidate indicators of root functional strategy, but not yet as operational measurements. These pathways are conceptually appealing, but their practical validity requires careful scrutiny. Several fundamental caveats warrant explicit discussion:

1. Multiple confounds on canopy temperature. A cooler canopy during drought does not necessarily indicate deeper rooting. Variation in stomatal regulation strategy (isohydric vs. anisohydric behaviour) can produce markedly different thermal signatures from trees with identical root systems. Leaf traits (size, orientation, pubescence), canopy structure (crown density, self-shading), atmospheric forcing (wind speed, vapour pressure deficit), and phenological state (deciduousness, leaf age) all influence canopy temperature independently of root function (Rowland et al., 2023). Unless these variables are measured or controlled, the inferential leap from canopy temperature to rooting depth is too large to support strong claims. We regard this as the central limitation of canopy-based root inference and emphasise it accordingly: it is a fundamental identifiability problem that multi-sensor fusion can only reduce, not eliminate — multiple distinct combinations of above-ground and below-ground traits can produce the same canopy thermal signature. Adding hyperspectral, SIF, and structural information narrows the set of consistent hypotheses, but does not, in general, yield a unique solution without additional ground-based constraints.
2. Above-ground change does not necessitate below-ground coordination. Changes in spectral indices or leaf area may reflect purely above-ground physiological responses (e.g., photoprotective pigment changes, premature leaf abscission) that are independent of, or only loosely coupled to, root-system adjustments. The assumption that above-ground and below-ground traits are tightly coordinated under stress is physiologically plausible but not universal across species or stress intensities. Some species may invest heavily in above-ground drought avoidance (e.g., deciduousness) with minimal root-system modification, while others may expand root systems without corresponding canopy changes (Fan et al., 2017).
3. The validation problem. Even if statistical associations between canopy signals and root traits are established, such correlations may not hold across sites, species, or stress scenarios. The critical question is: how can these inferences be validated? Possible validation strategies include (i) pairing UAV campaigns with destructive root sampling or isotope-based water sourcing measurements at a subset of trees; (ii) comparing aerial thermal monitoring with independent measurements of root-zone soil moisture depletion patterns from sensor networks; and (iii) leveraging existing long-term forest monitoring sites (e.g., FLUXNET, ICOS networks) where both canopy and below-ground variables are tracked. Each of these has operational limits that deserve explicit statement. Destructive root sampling in mature forests is ethically and logistically constrained, typically feasible for only a small number of trees per site, and mainly informative for structural traits such as rooting depth, root-to-shoot biomass ratio, and coarse architecture rather than dynamic functional properties; soil coring and whole-tree excavation also inevitably disturb the rhizosphere. Isotopic tracing (δ^2^H and δ^18^O) identifies the depth and source of water being taken up, but at limited temporal resolution—typically a handful of campaigns per season—with depth assignment dependent on site-specific soil-water isotope profiles and vulnerable to mixing and evaporative enrichment. Separating root water uptake from direct soil and surface evaporation in a soil-moisture time series is itself a non-trivial inverse problem, usually requiring combinations of soil-depth profiles, sap flow, eddy-covariance partitioning of evaporation and transpiration, or isotope-constrained models; any validation of root inference should therefore be understood as a constraint on plausibility rather than an unambiguous ground truth. Without rigorous validation against independent below-ground data, inferences from canopy signals remain hypotheses rather than operational phenotyping tools.
4. Scope limitation: drought is not the only stressor. The framework as presented is largely structured around drought stress and water-related root function. However, root systems are multifunctional: they acquire nutrients, form mycorrhizal associations, anchor trees structurally, store carbohydrates, and interact with the rhizosphere microbiome. Even if the link between aerial signals and root water uptake can be validated for drought scenarios, it is far less clear that other root functions — equally important for resilience to biotic stress, nutrient limitation, or wind loading — can be inferred from UAV data fusion. Future work must consider whether this framework generalises beyond the drought context or whether it represents a drought-specific analytical tool.

**Figure 4:**
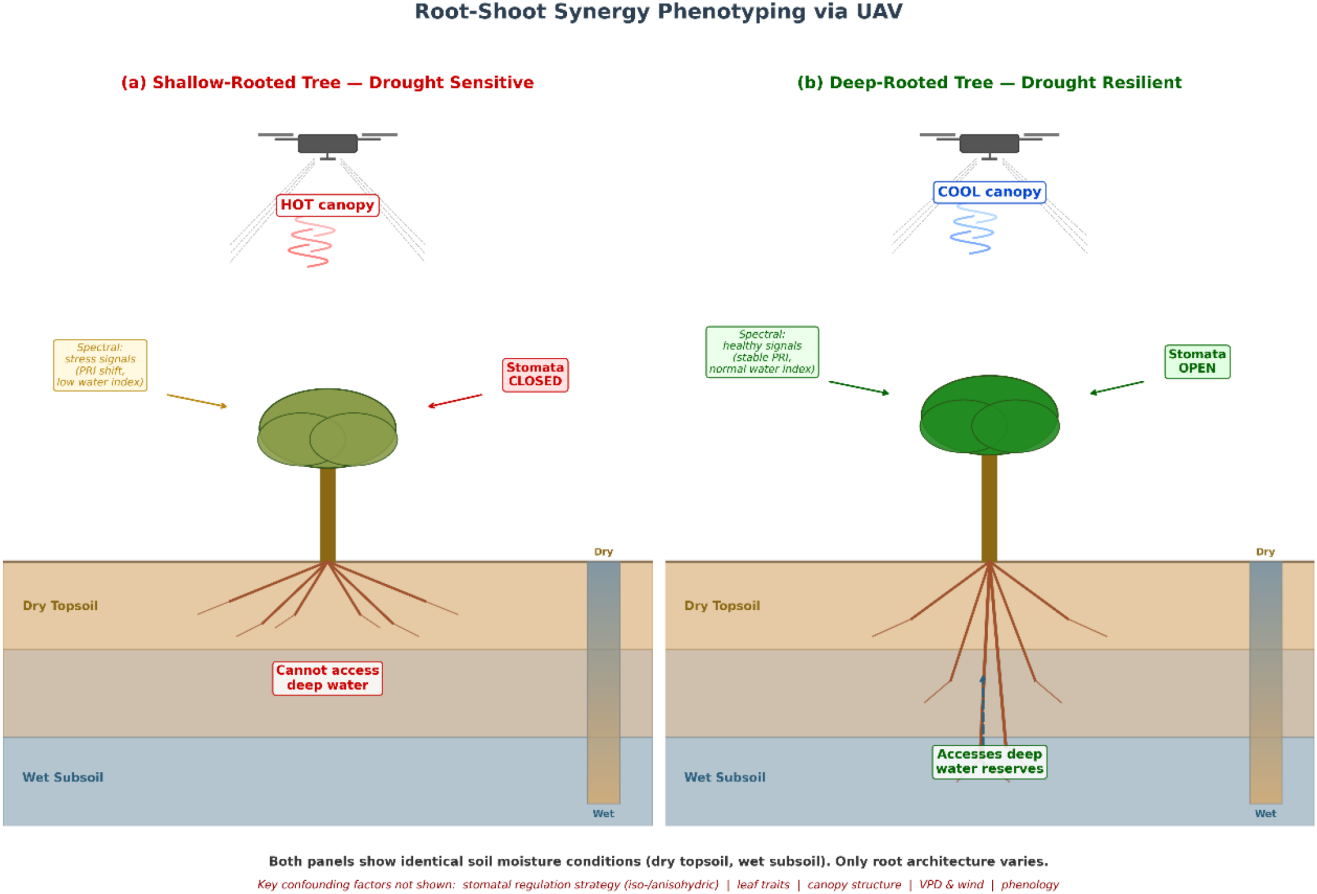
Conceptual framework of root-shoot synergy phenotyping via UAV. To isolate the effect of rooting strategy on canopy signals, the diagram holds soil water conditions constant while varying root architecture. Both panels show the same soil moisture profile (dry topsoil, wetter subsoil). Left panel: a shallow-rooted tree that cannot access deeper water, leading to stomatal closure, elevated canopy temperature (‘hot’ thermal signature), and spectral stress signals. Right panel: a deep-rooted tree accessing subsoil moisture, maintaining transpiration and presenting a cooler canopy with healthy spectral reflectance. Key confounding factors — stomatal regulation strategy, leaf traits, canopy structure, atmospheric conditions — are indicated alongside the main inferential pathways (dashed arrows), illustrating that the relationship between canopy signals and root function is mediated by multiple interacting variables.

The overall conceptual framework for root-shoot synergy phenotyping, illustrating both the proposed inferential pathways and the key confounding factors, is represented in **Figure 4**.

### 4.3 The role of modeling and AI in integration

Bridging the gap between a cool canopy pixel and a deep, functionally active root system requires sophisticated analytical frameworks. Functional-Structural Plant Models (FSPMs) simulate how specific root architectures and hydraulic properties influence above-ground canopy signals under varying soil moisture conditions (Mencuccini, 2003; Lobet et al., 2014). These models encode the biophysics of the SPAC, allowing researchers to test hypotheses about root function and generate predictions that can be compared against aerial observations. If, for a given set of environmental conditions, a model predicts that a 2-metre rooting depth should produce a particular canopy temperature trajectory during dry-down, and the observed thermal time-series deviates substantially, this constrains the plausible range of root traits for that individual tree.

AI adds a complementary, data-driven dimension. DL models capable of processing spatio-temporal data sequences — recurrent neural networks (RNNs), temporal CNNs, or attention-based architectures — can be trained to recognise patterns in multi-modal aerial imagery that are characteristic of specific below-ground root behaviours and water-acquisition strategies (Harfouche et al., 2019; Pieruschka and Schurr, 2019). The idea is to leverage the entire canopy as a ‘distributed sensor’ for the soil and root system, learning non-linear relationships that are analytically intractable.

This is an ambitious proposition, however, and must be assessed critically. Training such models requires paired datasets: high-throughput canopy observations linked to independent below-ground measurements at the same trees. As discussed in Section 4.1, such paired data are extremely rare in forest settings. Without adequate training data, models risk learning correlations that are statistically robust but biologically spurious — the classic problem of models being “right for the wrong reasons.” Transfer learning from agricultural settings, where root phenotyping is more tractable, might provide a partial solution, but the extrapolation from crop to tree root systems is itself uncertain given the vast differences in scale, longevity, and mycorrhizal associations.

The most promising path forward may be a hybrid approach that combines process-based modeling with data-driven AI. Physics-informed neural networks (PINNs) or hybrid model-data assimilation frameworks can embed known SPAC relationships as constraints, reducing the degrees of freedom that must be learned purely from data and thereby improving generalisation from limited training samples (Reichstein et al., 2019). For example, a model could be constrained to respect mass balance in the soil-root-stem-leaf water pathway, so that any inferred root water uptake must be physically consistent with observed canopy transpiration and measured soil moisture depletion. This approach does not eliminate the need for validation data, but it makes more efficient use of the limited data available.

### 4.4 Ground-based root sensing: capabilities and realistic constraints

A comprehensive assessment of the root-shoot integration framework must include a frank evaluation of ground-based root sensing technologies, which are often invoked as the validation complement to aerial observations.

Ground Penetrating Radar (GPR) can non-invasively map coarse root architecture (depth, distribution, diameter) without excavation (Barton and Montagu, 2004; Zou et al., 2020). Yet GPR performance is highly dependent on soil conditions: clay-rich, wet, or rocky soils attenuate the radar signal, substantially reducing detection capability. Critically, forest soils differ markedly from the agricultural soils in which GPR has been most extensively validated; high organic matter content, stone presence, and irregular soil horizons are the norm rather than the exception. Moreover, GPR detects dielectric contrasts between roots and surrounding soil, making no distinction between living and dead roots — a fundamental limitation for assessing functional root activity. The processing chain from raw radargrams to usable root architecture maps is also considerably longer and more uncertain than for above-ground remote sensing data, requiring substantial expertise and site-specific calibration.

Electrical Resistivity Tomography (ERT) and Electromagnetic Induction (EMI) offer the potential to image dynamic soil moisture changes around root zones, providing a functional view of water uptake patterns. These techniques are promising in principle, but deployment in forests faces practical constraints: electrode arrays must be installed and maintained *in situ*, limiting spatial coverage; measurements are typically point-based or along transects rather than area-wide; and the geophysical inversion from apparent resistivity to soil moisture distribution involves non-unique solutions that depend on assumed soil properties. As with GPR, these techniques do not directly measure roots — they measure soil properties that are influenced by root activity, among other factors.

A key question is whether these ground-based technologies can achieve ‘dynamic’ monitoring in a meaningful sense, as the title of this review implies. Moving heavy geophysical equipment through dense forest terrain is fundamentally different from deploying a UAV, and the temporal resolution achievable with ground-based geophysical surveys is measured in days to weeks, not the hours to days possible with aerial platforms. Permanent installations at fixed monitoring sites (e.g., permanent ERT transects at long-term ecological research stations) offer one path to higher temporal resolution, but at the cost of spatial coverage — the opposite trade-off from UAV-based sensing.

Is the root-shoot integration problem ultimately solvable? The honest assessment is that current technologies do not yet allow fully validated, high-throughput characterisation of root function from combined aerial and ground observations in forests. What they do offer is a set of complementary constraints — aerial data constraining above-ground physiology, ground-based geophysics constraining soil and root-zone properties, eco-hydrological measurements constraining water sources and uptake depths — that, when combined with process-based models, can progressively narrow the range of plausible root functional types for a given tree or stand. This incremental constraint-based approach, rather than a single definitive measurement, is likely the realistic near-term pathway.

## 5 Future perspectives

### 5.1 From data to decisions: the forest digital twin

A promising future direction for forest phenomics lies in the convergence of multi-sensor fusion, physiological modeling, and AI to create dynamic digital twins of forest ecosystems (Feng et al., 2021; Das, 2025). A digital twin — a concept first defined in Section 1 — goes beyond static 3D representation. It constitutes a dynamic, predictive virtual replica that continuously integrates real-time sensor data (from UAVs, satellites, ground-based IoT sensors) with process-based physiological and ecological models (Heilmeier, 2019; Smith et al., 2021). By feeding HTP-derived traits into these digital twins, researchers can simulate how different genotypes or forest stands will respond to future climate scenarios — specific temperature anomalies, prolonged drought events, compound extremes — and make proactive decisions on species selection, thinning regimes, and water management strategies before critical stress events occur (Harfouche et al., 2019; Xu and Jiang, 2025).

Realising this vision requires overcoming the data integration and below-ground sensing challenges discussed in Section 4. A digital twin that models only canopy processes, ignoring root dynamics and soil interactions, will produce systematically biased predictions of drought resilience. The eco-hydrological constraints discussed in Section 4.1 — soil moisture depletion patterns, isotope-traced water sources, remotely sensed root-zone moisture — provide pathways for incorporating below-ground information into twin frameworks, even where direct root measurements are unavailable.

### 5.2 Unlocking the underground: non-destructive root phenotyping at scale

While inferring root function from above-ground signals offers a pragmatic interim approach, the long-term goal remains direct, non-destructive quantification of root systems *in situ* at scale. GPR and ERT are becoming more sophisticated (Section 4.4), and their integration with aerial canopy platforms will eventually help bridge the root-shoot gap. However, realising ‘dynamic’ root monitoring in forests comparable to what aerial sensors achieve for canopies will require substantial technological advances beyond current geophysical capabilities.

Alternative or complementary approaches deserve consideration. High-resolution soil moisture sensor networks, when deployed at sufficient density, can reveal root water uptake patterns through inverse modelling of soil water flow (Feldman et al., 2023). Isotope-based water tracing can identify the soil depths and water sources accessed by individual trees under different stress conditions (Bachofen et al., 2024). While these methods are individually limited in spatial coverage, their combination with aerial observations and process-based models offers a multi-constraint approach to root functional phenotyping that may be achievable in the near term.

Ultimately, the development of standardised protocols, open benchmark datasets incorporating both above- and below-ground variables, and collaborative networks of instrumented forest sites will be essential for validating and operationalising the integrated phenotyping framework proposed in this review. To structure this agenda, we find it helpful to distinguish short-, medium-, and long-term priorities. In the short term (~1–3 years), the community can consolidate paired above- and below-ground datasets from existing long-term sites (e.g., FLUXNET, ICOS, ForestGEO, NEON), publish standardised multi-sensor acquisition protocols, and release initial open benchmarks. In the medium term (~3–7 years), these benchmarks should be used to evaluate hybrid physics-informed and data-driven models, develop routine uncertainty quantification (including predictive intervals and calibration diagnostics) for digital-twin outputs, and couple aerial campaigns with repeated isotope and geophysical measurements at a subset of sites. In the long term (~7–15 years), the goal is operational, multi-site digital twins with validated below-ground components and explicit uncertainty propagation from sensor to management decision. A useful open benchmark dataset would, at minimum, contain: co-registered UAV thermal, multispectral or hyperspectral, and LiDAR observations at sub-metre resolution; repeat campaigns across a full growing season including at least one drought episode; paired *in-situ* measurements of sap flow, leaf water potential, soil moisture profiles, and δ^2^H/δ^18^O of xylem and soil water; and ancillary metadata on stand composition, soil type, and weather forcing, released under open licenses with stable DOIs. Cost considerations also matter for operational forestry: a UAV thermal/multispectral campaign over a single stand is typically achievable for low thousands of USD per visit, whereas fully instrumented sites with sap-flow, soil-moisture, and isotope capacity remain considerably more expensive and are only realistic at a network of reference plots. Prioritising a small number of well-instrumented reference sites, with wider aerial coverage over production forests, is likely to be the most cost-effective near-term configuration.

## 6 Conclusion

The phenotyping bottleneck in forestry has fundamentally shifted. While hardware limitations remain important—particularly for below-ground sensing, where proximal geophysical tools still fall short of the throughput achieved by aerial platforms—the dominant bottleneck has increasingly shifted toward integration, interpretation, and validation. For the above-ground sensors and platforms required to detect early physiological stress in canopies are now largely available. The central challenge is coherently weaving multi-sensor data into a framework that connects aerial phenotypic signals to underlying root function and whole-plant physiology. This review has argued that bridging this gap requires acknowledging — not glossing over — the substantial assumptions involved in inferring below-ground traits from canopy observations, the multiple confounding variables that complicate such inferences, and the critical need for rigorous validation against independent below-ground data.

Progress will depend on several converging efforts: advancing multi-sensor fusion methods that combine thermal, spectral, fluorescence, and structural data into integrated physiological assessments; incorporating eco-hydrological constraints (soil moisture dynamics, isotope-traced water sources) into interpretive frameworks; developing hybrid modeling approaches that embed known SPAC biophysics into data-driven AI architectures; expanding ground-based geophysical sensing capabilities for forest soils; and fostering open science through standardised benchmark datasets and Explainable AI tools that make model predictions biologically interpretable. The specific contribution of this review is threefold: (i) to systematise the canopy-bias problem in forest HTP and make the identifiability issue explicit; (ii) to propose a constraint-based framework in which aerial, eco-hydrological, and process-based information jointly narrow the space of plausible root functional strategies, with clearly stated assumptions and validation requirements; and (iii) to articulate a staged research agenda—short-, medium-, and long-term—linking benchmark datasets, hybrid modelling, and uncertainty quantification to realistic operational deployment. By pursuing these directions, the forestry community can leverage HTP to accelerate the breeding and deployment of climate-resilient forests — forests equipped to sustain their ecological functions under the escalating pressures of a changing climate.
